# Coenzyme Q10 plus Multivitamin Treatment Prevents Cisplatin Ototoxicity in Rats

**DOI:** 10.1371/journal.pone.0162106

**Published:** 2016-09-15

**Authors:** Laura Astolfi, Edi Simoni, Filippo Valente, Sara Ghiselli, Stavros Hatzopoulos, Milvia Chicca, Alessandro Martini

**Affiliations:** 1 Bioacoustics Research Laboratory, Department of Neurosciences, University of Padua, Padua, Italy; 2 Foundation Onlus ‘Staminali e Vita’, Padua, Italy; 3 ENT surgery - Department of Neurosciences, University of Padua, Padua, Italy; 4 Clinic of ENT, University of Ferrara, Ferrara, Italy; 5 Department of Life Sciences and Biotechnology, University of Ferrara, Ferrara, Italy; University of PECS Medical School, HUNGARY

## Abstract

Cisplatin (Cpt) is known to induce a high level of oxidative stress, resulting in an increase of reactive oxygen species damaging the inner ear and causing hearing loss at high frequencies. Studies on animal models show that antioxidants may lower Cpt-induced ototoxicity. The aim of this study is to evaluate the ototoxic effects of two different protocols of Cpt administration in a Sprague-Dawley rat model, and to test in the same model the synergic protective effects of a solution of coenzyme Q10 terclatrate and Acuval 400^®^, a multivitamin supplement containing antioxidant agents and minerals (Acu-Qter). The Cpt was administered intraperitoneally in a single dose (14 mg/kg) or in three daily doses (4.6 mg/kg/day) to rats orally treated or untreated with Acu-Qter for 5 days. The auditory function was assessed by measuring auditory brainstem responses from 2 to 32 kHz at day 0 and 5 days after treatment. Similar hearing threshold and body weight alterations were observed in both Cpt administration protocols, but mortality reduced to zero when Cpt was administered in three daily doses. The Acu-Qter treatment was able to prevent and completely neutralize ototoxicity in rats treated with three daily Cpt doses, supporting the synergic protective effects of coenzyme Q terclatrate and Acuval 400^®^ against Cpt-induced oxidative stress. The administration protocol involving three Cpt doses is more similar to common human chemotherapy protocols, therefore it appears more useful for long-term preclinical studies on ototoxicity prevention.

## Introduction

Cisplatin (cis-diamminedichloroplatinum II) (Cpt) is a powerful antineoplastic DNA alkylating agent used to treat many types of solid tumours including testicular, ovarian, breast, lung, bladder, head, neck and uterine cervix carcinomas [1,2]. The Cpt is known to cause reversible and irreversible side effects on many organs including kidneys, bone marrow, gastrointestinal tract, brain and inner ear [1–4].

In animal models, the ototoxic effects of Cpt include morphological and functional changes of organ of Corti, stria vascularis and spiral ganglion, and mainly involve the loss of auditory outer hair cells (OHC) along a base-to-apex gradient, leading to a hearing loss mostly affecting the higher frequencies [4–9]. In patients receiving Cpt chemotherapy, ototoxicity is characterized by a loss of auditory hair cells, degeneration of the stria vascularis, a significant decrease in spiral ganglion cells [8], and an associated bilateral hearing loss at high frequencies (4–8 KHz) [4,7,8]. Clinical protocols may change according to the tumour but usually Cpt is administered in more doses, pure or in association with other drugs [1,2]. In animal models, the research protocols involve high doses that cause a high mortality within a week from treatment [7]. In order to evaluate long-term Cpt effects it is necessary to establish a protocol aimed to significantly decrease animal mortality.

The administration of antineoplastic agents during chemotherapy is known to increase oxidative stress and production of reactive oxygen species (ROS). A way through which ROS may cause cell damage and death is by lipid peroxidation [5,10,11]. Several studies dealt with the direct cytotoxic mechanisms of Cpt, involving DNA damage, mitochondrial dysfunction, depletion of glutathione and antioxidant enzymes, and cochlear injury with OHC loss. These damages are caused by production of reactive oxygen and nitrogen species, such as superoxide anion (O2^−^), and nitric oxide (NO) [12,13].

Several antioxidants have been tested *in vitro* or *in vivo* to prevent Cpt-induced ototoxicity [14–24]. Local inner ear administration of cytoprotective antioxidants such as D- or L-methionine [14–17], vitamin E [18,19], *Ginkgo biloba* extract [21] and thiourea [22] may provide protection against Cpt-induced loss of OHC and auditory function in rat or Guinea pig models. Systemic administration of L-methionine [23] or salicylate [24] significantly lowers Cpt ototoxicity in the rat inner ear.

Chemotherapy involves a reduction of plasma levels of antioxidants such as vitamin E, vitamin C, and β-carotene. Based on these studies, common antioxidants such as vitamin E (mixed tocopherols and tocotrienols), β -carotene (natural mixed carotenoids), vitamin C (ascorbic acid), and vitamin A (retinoic acid) are usually prescribed during chemotherapy [25]. In rats, vitamin administration is known to reduce Cpt ototoxicity by decreasing lipid peroxidation [26].

Coenzyme Q10 (2, 3-dimethoxy-5-methyl-6-decaprenyl-1, 4 benzoquinone, CoQ10) is involved in electron and proton transport in the mitochondrial respiratory chain. This antioxidant reacts with ROS and lipoperoxides, preventing damage of biomolecules in tissues and cell compartments [27–29].

The CoQ10 is not water-soluble and scarcely soluble in lipids, thus it is hardly bioavailable. Recently, a terclatrate version of the coenzyme (Coenzyme Q10 terclatrate, Q-TER^®^) with improved water solubility was developed [30,31]. The water solubility improves the activity of CoQ10 against oxidative injures, lipid peroxidation and mitochondrial damage [32,33]. The Q-TER^®^) is obtained by terclatration, a patented solid-state physical procedure (WO/2003/097012, Actimex Srl, Piacenza, Italy) in which the coenzyme (10% w/w) is added to a carrier and a catalyst [34].

The aim of the present study was to evaluate in a Sprague-Dawley rat model whether the administration of Cpt in three consecutive doses may cause the same hearing loss as that caused by one cumulative dose, and whether an association of Acuval 400^®^ and coenzyme Q10 terclatrate (Acu-Qter) may exert protective effects against Cpt-induced ototoxicity in the same model.

## Materials and Methods

### Animals and treatment groups

Thirty-three male Sprague Dawley rats (150–200 g; Charles River, Milan, Italy) were used in this study. The animals were treated in strict accordance with the recommendations of the DL 116/92 Italian guidelines with reference to European Economic Community directive 86–609. All experiments were approved by the Ethics Committee for Animal Usage of the University of Ferrara (Ferrara, Italy), registration no. 15599. The animals were randomly divided in the following groups:

- A, untreated controls (n = 5)
- B, controls orally treated for five days with a daily dose of 100 mg/kg of Acuval 400^®^ and 500 mg/kg coenzyme Q10 terclatrate, dissolved in water (Acu-Qter) (n = 5);
- C, animals treated intraperitoneally with a single dose of cisplatin (Cpt) 14 mg/kg (n = 7);
- D, animals orally treated with Acu-Qter as group B and intraperitoneally with a single Cpt dose (14 mg/kg) on the second day, 2 hrs after Acu-Qter administration (n = 6);
- E, animals intraperitoneally treated with Cpt 4.6 mg/kg/day for three days (n = 5);
- F, animals treated with Acu-Qter as group B and intraperitoneally with Cpt 4.6 mg/kg/day from day 2 to day 4, 2 hrs after Acu-Qter administration (n = 5).

### Drugs

The Q-TER^®^ was manufactured by Pharmaland (Republic of San Marino, Italy) using an industrially available native CoQ10 (Kaneka Pharma Europe, Brussels, Belgium). Acuval 400^®^, a dietary multivitamin supplement containing vitamins A, E, B1, B2, B6 and B12, L-Arginine, *Ginkgo biloba* extract, minerals (Mg, Se, Zn) and a small amount of Q-TER^®^ (0.31 g/100 g), was provided by Scharper Healthcare Srl. (Milan, Italy). Cisplatin (1 mg/ml) was obtained from Ebewe Pharma GMBH (Unterach, Austria). Oral administrations were performed using a feeding tube. For the ABR recordings, animals were pre-treated with an anaesthetic solution composed by 50 mg zoletil 100 (Virbac, Milan, Italy) dissolved in 1 ml physiological saline solution, added with 0.5 ml 2% xylazine (Bayer Animal Health, Mississauga, Ontario, Canada).

### Auditory brainstem responses

Auditory brain responses (ABR) were used to assess the auditory threshold as previously described [31]. All ABR testing was performed in a 1-m^3^ soundproof chamber with the animals placed on a homoeothermic blanket to maintain a constant body temperature at 37.5° Celsius. The ABR responses were recorded with 3 platinum-iridium needle electrodes placed sub-dermal over the vertex (positive), the mastoid (negative), and the dorsum area (reference/ground) of the animal. A tweeter sound transducer (flat response ±1.5 dB; 4.0–35 kHz) was used and a sound delivery plastic speculum was inserted into the external ear canal. The ABR was amplified 20000 times and filtered from 20 to 5000 Hz. Each recording was the average of 1000 individual responses. The ABR was generated in response to 2, 4, 8, 16, 32 kHz tone pips (1-ms rise-fall time, 10-ms plateau) from 100 to 30 dB in 5-dB intervals. Threshold was defined in two averaged runs as the lowest intensity at which a measurable wave III ABR was observed. Earplugs were used to occlude the contralateral ear to avoid possible binaural stimulation at the highest stimulus intensities.

In all animals the auditory function was assessed by ABR at the beginning (day 0) and at the end (day 5) of treatment. The general health conditions and the body weight of each animal were monitored daily. The initial average weight of rats was 184 ± 18 g and the difference in weight within each group was not significant. After day 5 or earlier (if significant distress signs such as unusual bleeding and dyspnea appeared), the animals, anesthetized with zoletil and xylazine as previously described, were painlessly sacrificed by decapitation.

### Statistical analysis

The average, standard deviation and standard error of ABR threshold shifts, expressed in sound pressure level decibel (dB SPL) for each experimental groups were calculated at day 0 and at day 5. The STATISTICA 7.1 software (StatSoft Inc., Padua, Italy) was used to calculate the basic statistic parameters, the paired t-test, ANOVA one way (Bonferroni test) and the non-parametric Wilcoxon Signed-Ranks Test and correlation indexes. Statistical significance was set at p-value < 0.05.

## Results

The ABR thresholds were detected from both ears at day 0 and the averages obtained from all the animals (expressed in dB SPL) are reported in Table 1. No significant differences were detected thus the rats were randomly divided in six groups as previously described.

**Table 1:** ABR threshold averages, expressed as sound pressure level decibel (dB SPL) at day 0.

Frequencies	dB SPL	St. Dev.	F	p-value
Click	28	5	1.283	0.285
2 kHz	64	7	2.361	0.103
4 kHz	47	7	0.860	0.428
8 kHz	40	8	1.840	0.168
16 kHz	34	7	1.000	0.374
32 kHz	48	6	1.256	0.292

St. Dev.: standard deviation; F: One-way ANOVA

### Cisplatin ototoxicity treatments

The mortality ratio when Cpt was administered in one dose was 28% in group C and 17% in group D (pre-treated with Acu-Qter) (Table 2). In groups intraperitoneally treated with Cpt 4.6 mg/kg/day for three days, without (E) and with (F) Acu-Qter pre-treatment, the mortality ratio was zero.

**Table 2:** Cisplatin toxicity expressed as mortality ratio and weight variations.

Group	Mortality ratio (%)	Weight variation			
		Δg	Δ%	Z	p-value
A	0	22±9	12±6	2.023	0.043
B	0	24±2	14±1	2.201	0.043
C	28	-20±16	-9±7	1.992	0.018
D	17	-27±19	-12±8	2.367	0.046
E	0	-40±7	-16±3	2.023	0.028
F	0	-35±9	-12±3	2.023	0.043

Δg: weight variation (g) ± standard deviation; Δ%: weight variation (percentage) ± standard deviation; Z: value of non-parametric Wilcoxon Signed-Ranks Test.

Comparing the body weight of day 0 to day 5, a significant decrease was noticed in all Cpt-treated groups, while the control rats (groups A and B) significantly gained weight (p<0.01). The body weight loss was not significantly different among Cpt-treated groups (p>0.05), but was significant in comparison to control groups (p<0.05) (Table 2).

**Fig 1:**
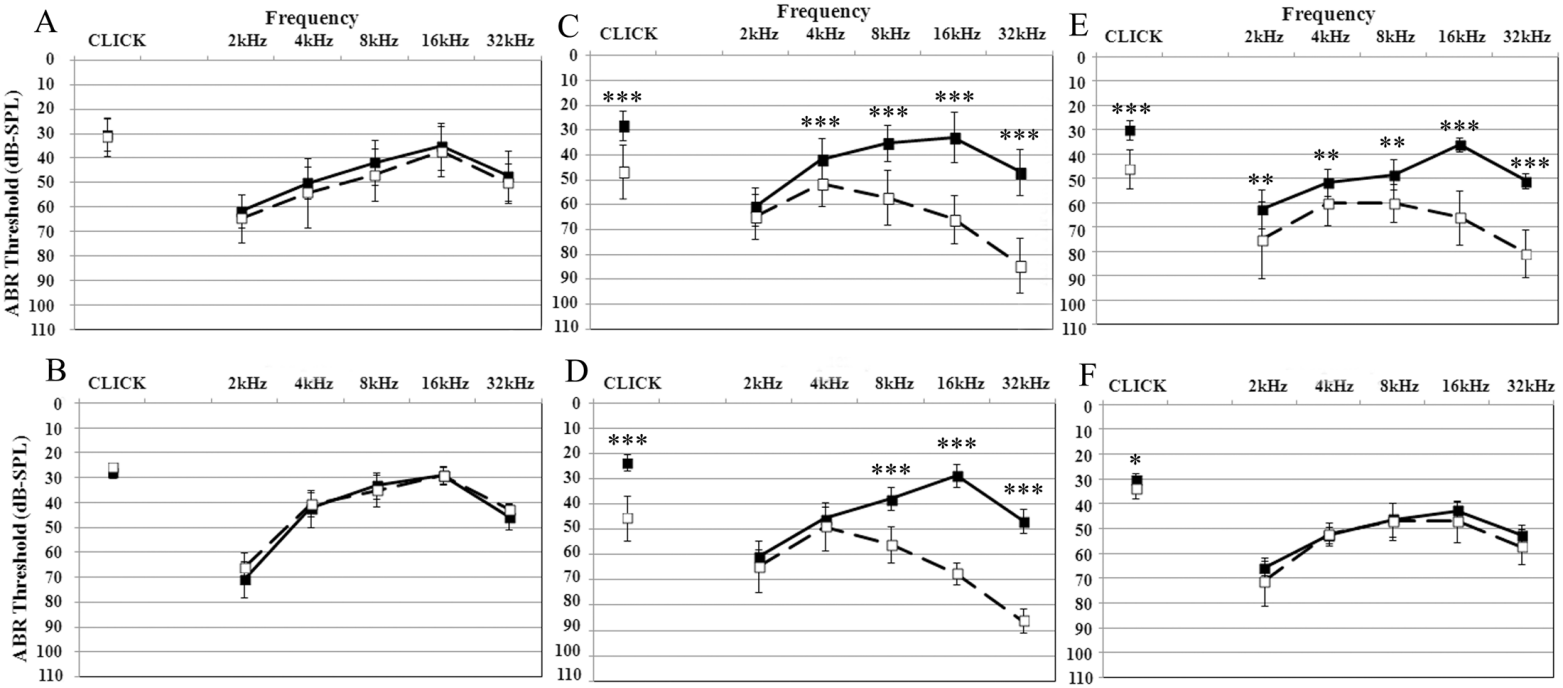
ABR threshold averages for each frequency measured before (continuous line) and 96 h after (dashed line) cisplatin treatment. The letters A-F indicate the treatment group described in the Materials and Methods section. ABR thresholds are expressed in dB SPL ± standard deviation. **: t test p-value<0.01; ***: t test p-value<0.001.

**Fig 2:**
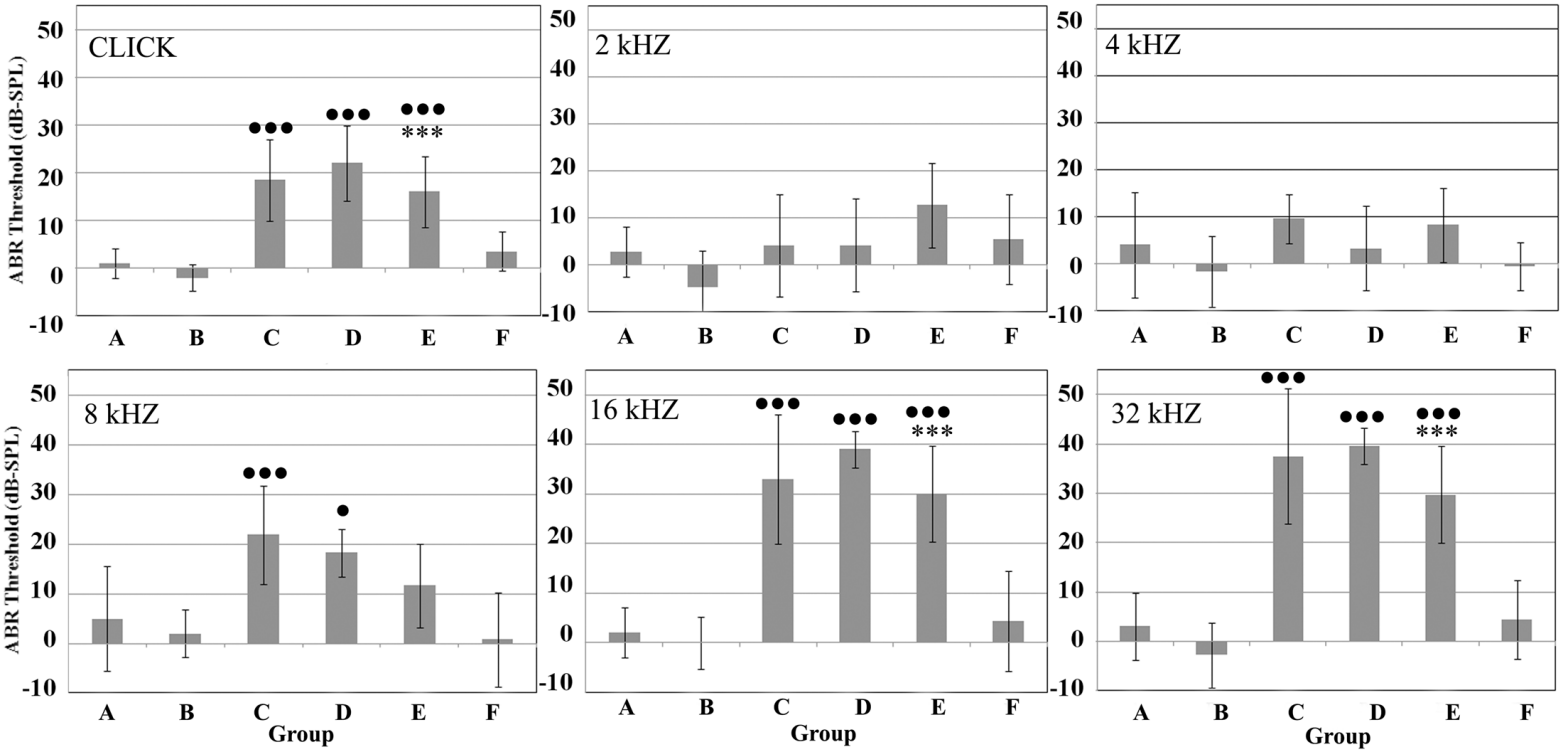
ABR threshold shifts for each treatment group (A-F) at different frequencies. ABR thresholds are expressed in dB ± standard deviation. ANOVA p-value significance in all groups of treated animals versus untreated controls (A): ● <0.05; ●● <0.01; ●●● <0.001; ANOVA p-value significance between groups untreated and treated with Acu-Qter (A and B, C and D, E and F): *** <0.001.

The ABR threshold and threshold shift averages of all experimental groups at day 0 and day 5 are respectively reported in Figs 1 and 2. In Fig 1, both Cpt-treated groups (C and E) showed a significant hearing loss (p<0.01), except at 2 kHz in group C (p>0.05). The threshold shifts reported according to frequencies in Fig 2 confirm the significant hearing losses at click and at higher frequencies in group C and E in comparison to controls.

### Hearing loss prevention

Control animals orally treated for five days with a daily dose of Acu-Qter (group B) maintained the normal hearing without adverse effects (Fig 1). Animals treated with Acu-Qter and a single Cpt dose (group D) showed a significant hearing threshold elevation at all frequencies except at the lower ones (2 and 4kHz) (p>0.05) (Fig 1). On the contrary, the animals treated with Acu-Qter and three Cpt doses (group F) did not show any hearing loss at all frequencies (Fig 1). The threshold shift of group D shows a significant hearing loss at click and at the higher frequencies in comparison to controls (group A) (Fig 2). No significant differences were detected in hearing loss between group C and group D, both treated with one Ctp dose, respectively without and with Acu-Qter (Fig 2). A significant difference in hearing loss was however detected between group E and group F, both treated with three Cpt doses, respectively without and with Acu-Qter (Fig 2).

## Discussion

Cisplatin (Cpt) is a widely used chemotherapeutic agent, but its use is limited by dose-related side effects, among which there is ototoxicity, leading to irreversible sensorineural hearing loss [2–4]. In studies of Cpt ototoxicity a high mortality ratio is frequently reported in animal models, basically undermining long-term *in vivo* protocols.

Our results show that in rats a single Cpt dose of 14 mg/kg caused a 28% mortality ratio after 4 days, reduced to 17% in animals pre-treated with Acu-Qter. In both groups, the mortality ratio reduced to zero when the same Cpt amount was administered in three daily doses. These results confirm those previously reported in mice, in which a single Cpt dose of 15 mg/kg caused a 70% mortality after ten days, but only a 10% mortality when it was divided in 4 daily injections [7].

Another relevant side effect of Cpt toxicity is body weight loss (BWL), therefore in animal studies this parameters is always monitored in Cpt protocols in animal studies. In mice treated with 16mg/kg Cpt, a significant BWL occurred in both groups treated in one or in four doses [35]. In rats treated with 16mg/kg Cpt in a single dose, a 15–20% BWL was recorded [16], and a 10% one resulted when the same Cpt dose was administered in two separate cycles of 2mg/kg for 4 days [36]. When rats were treated with a higher Cpt dose (8 mg/kg for three consecutive days) the BWL increased to 20–40% [37].

In our experiments, when a dose of 14 mg/kg Cpt was administered in a single dose, the BWL after four days was 9% and increased to 16% when it was administered in three daily doses. These data support the fact that the effects of Cpt on BWL do not depend on its dosage protocol in one or more amounts, but on the total quantity administered [16, 35–37].

Concerning the effects of CoQ10, in a previous study on rats the significant BWL in animals treated intraperitoneally with Cpt (a double weekly dose of 2 mg/kg for 8 weeks) was recovered when the same animals were pre-treated with a combination of CoQ10 (10 mg/kg/day, intraperitoneally) and monosodium glutamate (500 mg/kg/day, orally) [38]. On the contrary, we did not detect a significant recovery of BWL when rats treated intraperitoneally by Cpt were orally pre-treated with Acu-Qter. A possible explanation is the different way of administration of CoQ10.

The Cpt-induced hearing loss is known to be caused by hair cell death, decreasing along the cochlea from the base to the apex [7,9]. With increasing dosage the hair cell loss increases, followed by damages to stria vascularis and collapse of Reissner’s membrane [4,5]. Our data agree with these observations because significant threshold shifts were detected at high frequencies (16 and 32 kHz). Moreover, when Cpt was administered in a single dose, a significant hearing loss also occurred at 8 kHz.

No significant effects on mortality ratio, BWL and ABR were detected in rats treated only with Acu-Qter. A significant protective effect of Acu-Qter was detected in ABR only in animals treated with three daily doses of Cpt.

Cisplatin is known to induce oxidative stress through lipid peroxidation and depletion in antioxidant enzymes leading to ROS accumulation in cochlear tissues [11]. The CoQ10 quenches ROS production and accumulation by interfering with the production of lipid peroxyl radicals (Bentinger et al., 2007), and by lowering the expression of NADPH oxidase [29] and NO production excess [30].

In our model, the Acu-Qter was able to counteract the cochlear damages when Cpt was administered in three daily doses and not in a single one. The inability of Acu-Qter to prevent ototoxicity when Cpt was administered in a single dose could be due to the fact that damages of cochlear structures induced by a single Cpt dose are so severe [4,7,35] that other mechanisms could be involved besides oxidative stress [39].

In human clinical protocols, Cpt is usually administered in several treatment sessions [1,2] and hearing impairment is directly correlated to high cumulative doses [2,40]. Our results show that our rat model is more similar to these protocols, thus better suitable for clinical trials.

In our rat model, Acu-Qter was able completely quench Cpt ototoxicity, whereas in previous *in vitro* and *in vivo* studies using antioxidants and anti-inflammatory drugs a complete neutralization of Cpt toxicity was never achieved [5,17–26,41–44].

The oral treatment with Acu-Qter did not affect the body weight loss caused by Cpt, thus Acu-Qter acted only locally in the cochlea and did not show systemic interactions. However, in order to test whether these interactions may occur, further investigations are required in rat cancer models.

## Conclusions

The results of the present study show that in the rat model the administration of cisplatin in three doses causes the same ototoxicity of a single dose, but with a significant decrease of mortality. This Cpt administration protocol is more similar to those employed in human chemotherapy, therefore it is more suitable for clinical trials and could also be useful for long-term Cpt studies on animal models.

A solution of coenzyme Q10 terclatrate associated to the multivitamin supplement Acuval 400^®^ (Acu-Qter) is able to neutralize ototoxicity when the same Cpt cumulative dose is administered in several smaller doses similar human clinical protocols. The Acu-Qter solution could therefore be employed to prevent Cpt ototoxicity induced by oxidative stress. Further studies are required on molecular mechanisms underlying the antioxidant effects of Acu-Qter and on its systemic interactions with Cpt in rat cancer models.
