# Correction: Coenzyme Q10 plus Multivitamin Treatment Prevents Cisplatin Ototoxicity in Rats

**DOI:** 10.1371/journal.pone.0185525

**Published:** 2017-09-21

**Authors:** Laura Astolfi, Edi Simoni, Filippo Valente, Sara Ghiselli, Stavros Hatzopoulos, Milvia Chicca, Alessandro Martini

In the Introduction, the sentence "The Q-TER^®^ is obtained by terclatration, a patented solid-state physical procedure (WO/2003/097012, Actimex Srl, Piacenza, Italy) in which the coenzyme (10% w/w) is added to a carrier and a catalyst [34]," the word “Piacenza” should be replaced with "Trieste."

In the Drugs sub-section of the Materials and Methods section, the first sentence should read: The Q-TER^®^ was provided by Scharper S.p.A. (Milan, Italy). Acuval 400^®^, a dietary multivitamin supplement containing vitamins A, E, B1, B2, B6 and B12, L-Arginine, Ginkgo biloba extract, minerals (Mg, Se, Zn) and a small amount of Q-TER1 (0.31 g/100 g), was provided by Scharper S.p.A. (Milan, Italy).

There is an error in the Funding section. The correct funding information is as follows: The work was supported by the Scharper S.p.A. (Milan, Italy). The funders had no role in study design, data collection and analysis, decision to publish, or preparation of the manuscript.

There is an error in the Competing Interests section. The correct Competing Interests statement is: The work was supported by the Scharper S.p.A. (Milan, Italy). This does not alter our adherence to PLOS ONE policies on sharing data and materials.

There is an error in the Acknowledgements section. This section should read: We are grateful to Dr. G. Manini and Dr. D. Bellini for providing us the ACUVAL400^®^ and the Q-TER^®^ (Scharper, S.p.A.). The authors owe thanks also to Dr V. Cascella and Dr. P. Giordano for helpful discussion on the first draft of the manuscript.
